# Six-month effects of integrative treatment, therapeutic acupuncture and conventional treatment in alleviating psychological distress in primary care patients - follow up from an open, pragmatic randomized controlled trial

**DOI:** 10.1186/1472-6882-14-210

**Published:** 2014-06-30

**Authors:** Tina Arvidsdotter, Bertil Marklund, Charles Taft

**Affiliations:** 1 Institute of Health and Care Sciences, Sahlgrenska Academy, University of Gothenburg, Gothenburg, Sweden; 2 Department of Primary Health Care, University of Gothenburg, Gothenburg, Sweden; 3 Primary Health Care Research Development and Education Centre, Vänersborg, Sweden; 4 University of Gothenburg Centre for Person-Centred Care, Sahlgrenska Academy, University of Gothenburg, Gothenburg, Sweden

**Keywords:** Acupuncture, Anxiety, Depression, Integrative treatment, Person-centred care, Primary care, Psychological distress, Quality of life, Sense of coherence

## Abstract

**Background:**

To evaluate and compare 6-month effects of 8 weeks of an integrative treatment (IT), therapeutic acupuncture (TA), and conventional treatment (CT) in reducing symptoms of anxiety, depression and in improving health-related quality of life (HRQL) and sense of coherence (SOC) in psychologically distressed primary care patients.

**Methods:**

Patients who had participated in an open, pragmatic randomized controlled trial were followed up six months after treatment. The study sample consisted of 120 adults (40 per treatment arm) aged 20 to 55 years referred from four different primary health care centres in western Sweden for psychological distress. Assessments were made at baseline after eight weeks and after 24 weeks. Anxiety and depression were evaluated with the Hospital Anxiety and Depression scale (HADS), HRQL with the SF-36 Mental Component Summary scores (MCS) and SOC with the Sense of Coherence-13 questionnaire.

**Results:**

No baseline differences were found between groups on any outcome variable. At 24 weeks, IT and TA had significantly better values than CT on all variables. All three groups showed significant improvements from baseline on all variables, except HAD depression in CT; however, improvements were significantly greater in IT and TA than in CT. IT and TA did not differ on any outcome variable. Effect sizes were large in IT and TA for all variables and small or moderate in CT. Improvements on all variables seen after 8-weeks of IT and TA remained stable at 24 weeks and the CT group improved on HAD anxiety.

**Conclusions:**

IT and TA seem to be more beneficial than CT in reducing anxiety, depression, and in improving quality of life and sense of coherence after 24 weeks of follow up in patients with psychological distress. More research is needed to confirm these results.

**Trial registration:**

ISRCTN trial number NCT01631500.

## Background

Psychological distress is a prevalent mental health problem in the community [1-3], with reported rates of up to 38% [4]. Primary care provides the first level of mental health care within the formal health care system and most patients suffering from psychological distress are treated solely at this level [5-8]. Common treatment approaches for psychological distress in primary care include watchful-waiting, self-help training, exercise, various psychological therapies and pharmacological treatments. The effectiveness and appropriateness of these approaches is, however, debated, particularly for mildly distressed patients [9,10] and also for more severe cases [11].

Current guidance emphasizes the importance of taking into account patient preferences for treatment [12] and patient participation in clinical decision making has been identified as an important factor for improving treatment adherence and clinical outcomes [13]. Patients with psychological distress often prefer alternative forms of treatment [9,10] and the use of complementary and alternative medicine (CAM) modalities for managing psychological distress is widespread in the population [14]. Moreover, people with psychological distress are more likely to use CAM therapies than conventional medical or mental health treatments [15], largely due to dissatisfaction with such treatments [16]. There is therefore a need to further evaluate the evidence base for alternative treatments and to compare their potential benefits relative to those of usual care.

Acupuncture is one of the most widely used CAM therapies [17] and is a method of choice among individuals who tend to be resistant to conventional medicine [18]. Moreover, a number of systematic reviews have suggested that acupuncture may be a promising treatment option for reducing anxiety [19-21] and depression [14,22-24]. Nonetheless, a Cochrane review pointed to the need for comparative, longer term studies to assess the benefits of acupuncture relative to other interventions and to usual care [23]. Recently, in response to this call a randomized controlled trial was conducted in primary care settings comparing acupuncture, counseling and usual care [15]. That study reported significant three month reductions in depression after acupuncture compared to usual care (although not compared to counseling), and interestingly, these reductions were maintained at 12-month follow up [15].

We have previously assessed short term effects of integrative treatment combining therapeutic acupuncture with structured salutogenic dialogue or therapeutic acupuncture with non-directive dialogue, and usual care in alleviating psychological distress in primary care patients in a pragmatic randomized controlled study [25]. Our study showed that both the acupuncture and integrative treatments were effective in reducing anxiety and depression, and more effective than usual primary care treatment. Yet unpublished results from the same study indicate that the first two treatments were also associated with improved health-related quality of life (HRQL) and sense of coherence (SOC). The present study reports results from a 6-month follow up of the patients included in that study with respect to anxiety, depression, HRQL and SOC.

### Aim

The aim was to evaluate intermediate term effects (24 weeks) of eight weeks of integrative treatment (IT) vs. therapeutic acupuncture (TA) vs. conventional treatment (CT) on depression, anxiety, HRQL and SOC in primary care patients with psychological distress.

## Method

This study reports a six-month follow-up of patients who had participated in a pragmatic randomized controlled study comparing CT, TA and IT. Study participants, randomization procedures, interventions and data collection methods are described in detail elsewhere [25]. Briefly, the study was conducted between 2010–2011 at four primary health care centers in western Sweden after approval by the Regional Ethical Review Board, Gothenburg Sweden (Dnr: 365–08).

### Participants

**Figure 1:**
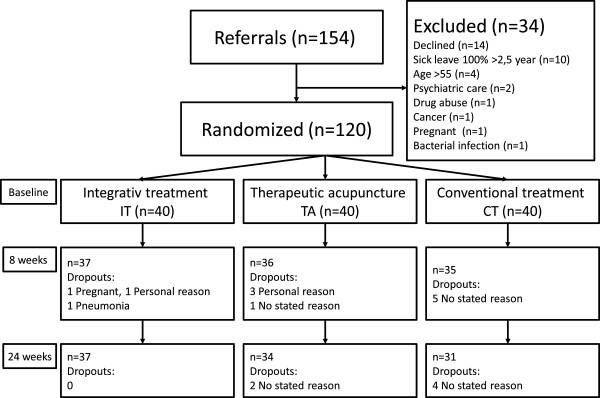
Flowchart of the patients in the study.

In total, 154 patients aged 20–55 years were initially recruited through referrals from primary care for complaints of psychological distress. Exclusion criteria were full sick leave >2.5 years, pregnancy, cancer, personality disorders, substance or alcohol use disorders and severe depression. Eligible referrals were contacted by telephone and mailed written information about the study, a written consent form and self-assessment questionnaires. Patients were thereafter scheduled for a one-hour visit at their primary health care center where a research nurse reviewed their medical histories and patients were asked to describe their illness experience. In total, 120 persons met inclusion and exclusion criteria and were included in the study. Patients were subsequently randomly allocated to one of the three treatment regimens using a simple randomization procedure (shuffled deck of cards) (Figure 1) (Table 1).

**Table 1:** Patient sociodemographic characteristics by Integrative Treatment (IT), Therapeutic Acupuncture (TA) and Conventional Treatment (CT)

**Variable**	**IT n = 40**	**TA n = 40**	**CT n = 40**
Sex: female	34 (85%)	32 (80%)	35 (88%)
Mean age (SD)	41 (8.1)	41 (9.0)	40 (9.1)
Education: High school	26 (65%)	28 (70%)	23 (58%)

Eighty-four percent (n = 101) of the patients were women, the average age was 41 and 65% had at least a high school degree. Primary diagnoses were: depression (30%), anxiety or panic disorders (20%), severe stress (20%), somatic symptoms/pain (20%) and sleep disorders (10%).

### Interventions

#### ***Therapeutic acupuncture***

Therapeutic acupuncture (TA) [26] was performed once a week (45-minute sessions) for eight consecutive weeks. Between two and 12 needles were inserted until a de qi sensation was achieved. The acupuncturist conversed freely with the patient about his/her condition and suggested lifestyle changes and relaxation methods.

#### ***Integrative treatment***

Integrative treatment (IT) combined TA with a person-centred [27-29], salutogenic dialogue inspired by Antonovsky’s salutogenic model [30]. The focus of the dialogue was to help the patients to become aware of and mobilize their strengths and potentials for coping with their condition. Areas discussed included inner feelings, personal relations, everyday activities (diet, exercise, relaxation, sleep habits) and existential issues. IT was performed once a week (60-minute sessions) for eight consecutive weeks.

#### ***Conventional treatment***

Conventional treatment (CT) consisted of usual care provided at each primary care center. Treatments included watchful waiting, pharmacological, psychological, psycho educational therapies or combinations thereof.

All patients were advised to continue their medication regimens, but patients in the TA and IT groups were asked not to begin psychological treatments or physiotherapy during the study period. TA and IT treatments were performed by the same experienced therapist (nine years’ clinical experience of salutogenic dialogue inspired by Antonovsky’s sense of coherence theory [30] and nine years as a certified TCM acupuncturist).

### Data collection and assessment instruments

Baseline self-assessment questionnaires were returned at the initial visit and follow-up questionnaires were returned by mail. Primary outcomes were anxiety and depression as measured by the Hospital Anxiety and Depression Scale (HAD). [31] The HAD is a 14-item questionnaire with seven items measuring anxiety (HAD-A) and seven measuring depression (HAD-D). Ratings are summed to give a score range of 0–21 for anxiety and depression, respectively, where higher scores indicate greater depression or anxiety. Scores between 8 and 10 represent possible cases of anxiety or depression and scores of 11 or above correspond to probable cases [32]. The validity and reliability of the Swedish version of the HAD has been shown to be good [33].

Mental health status was assessed with the Swedish version of the Short Form-36 (SF-36) [34]. The SF-36 is a 36 item, generic questionnaire measuring self-reported health status in eight domains: physical functioning, role limitations physical, bodily pain, vitality, general health, social functioning, role limitations emotional and mental health. Domain scores may be aggregated and normalized using a standard algorithm into two summary component scores, Mental Component Summary (MCS) and Physical Component Summary (PCS), where a value of 50 represents the population norm and higher scores indicate better health status. Only MCS scores were selected for evaluation to minimize multiplicity in the analyses and since physical health domains were considered less germane to the interventions.

Sense of coherence (SOC) [30] comprises three components: comprehensibility, manageability and meaningfulness. These concepts are relevant to how people manage different situations. If the person finds a situation comprehensible, manageable and meaningful, it is conceived as less stressful. The instrument consists of 13 questions which are rated on a seven-point scale. Low scores indicate a weak SOC and that the individual may require assistance in finding new strategies to deal with stressful situations. Normative values are not available for SOC scores [30,35]. The Swedish version of the SOC-13 scale has demonstrated adequate validity and reliability, with Cronbach’s alpha ranging from 0.70 to 0.92 [36,37].

### Statistical analyses

Data was analyzed on an intention-to-treat basis. Descriptive statistics characterized socio-demographic, clinical and outcome variables at baseline and follow up in each treatment group. Baseline between-group differences in gender and education level were assessed with the Chi^2^ test and age was evaluated with a one-way ANOVA. Between-group differences on outcome variables at baseline, after eight weeks of treatment and after 24 weeks from baseline, as well as change scores (baseline - 8 weeks, baseline - 24 weeks, and 8–24 weeks) were assessed with the Kruskal-Wallis test, followed by pairwise comparisons with the Mann–Whitney *U* test. Friedman’s test was used to test for within group differences between the three measurement points, followed by Wilcoxon signed rank test between two points. Non-parametric methods were used due to the skewed distribution and ordinal-level of the outcome data. Scores for missing questionnaires were imputed as the treatment group mean. Bonferroni correction was used to compute adjusted p-values for multiple comparisons. All tests were two-tailed and a 5% significance level was used throughout. All analyses were conducted using PASW SPSS version 18 (Chicago, Il) [38].

The clinical significance of change was assessed using effect sizes (ES). Effect sizes (ES) were calculated to estimate the magnitude of both the within-group changes in HAD-A and D, SF-36 MCS and SOC values between baseline and 24-week follow up and between group differences at 24-week follow up. Within-group ES was calculated as the difference between mean values divided by the standard deviation of change scores. Between-group ES was calculated as the difference between mean values divided by the pooled standard deviation. 95% confidence intervals for ES were calculated. ES magnitudes were interpreted against the criteria suggested by Cohen: trivial (0 to <0.2), small (≥0.2 to <0.5), moderate (≥0.5 to <0.8) and large (≥0.8) [39].

## Results

**Figure 2:**
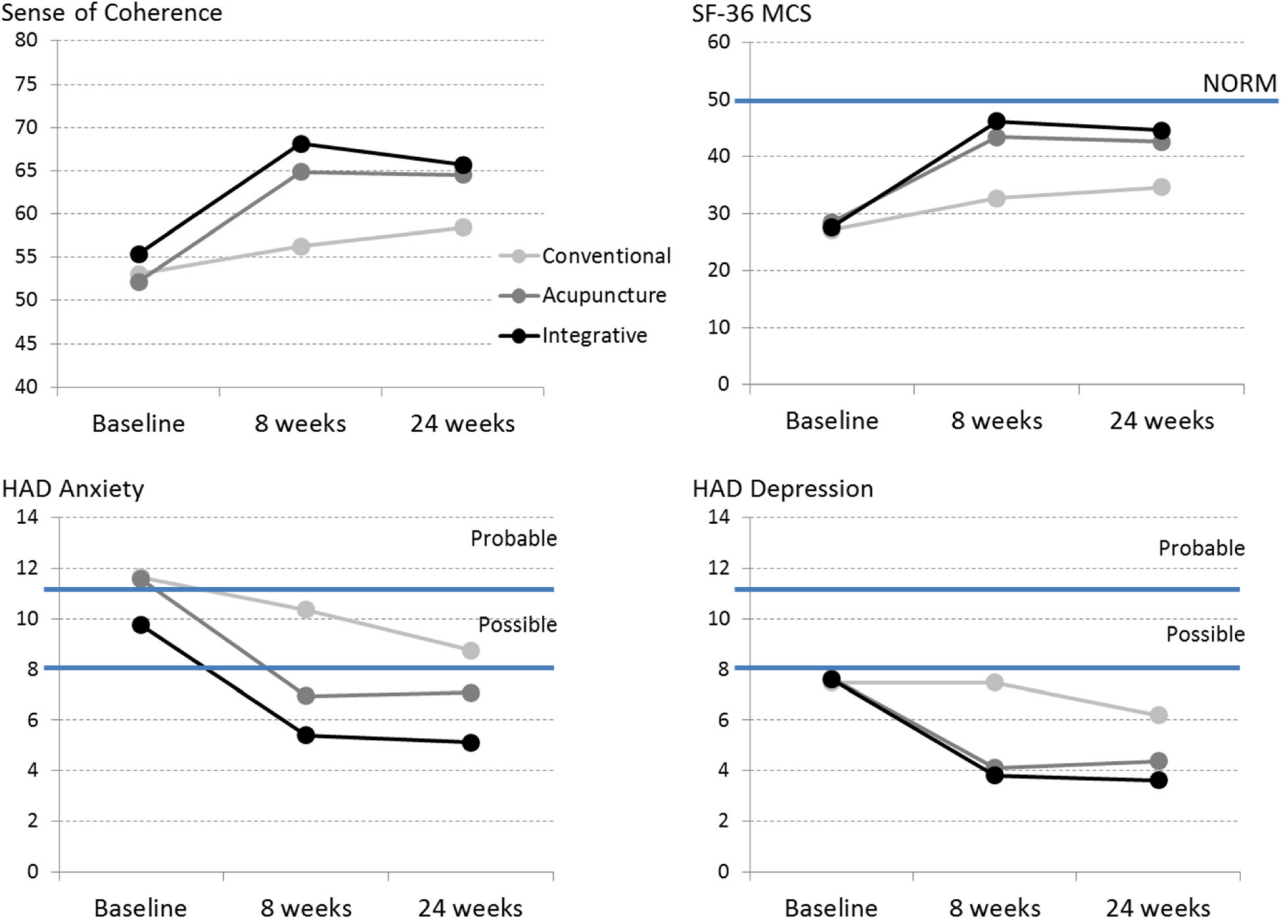
**HAD anxiety, HAD depression, SF-36 MCS and Sense of Coherence mean scores at baseline, and at 8 and 24-week follow up for the three treatment groups.** Solid horizontal lines indicate HAD cutoffs for possible (scores 8- < 11) and probable (≥11) and the SF-36 MCS norm value (50) for the general Swedish population.

Of the total 120 randomized participants, 102 (85%) returned questionnaires at 24-week follow up. Of these, 37 were in the IT group (86% women), 34 in the TA group (83% women) and 31 in the CT group (95% women). 12 of the 18 non-compliers completed eight weeks of treatment (Figure 1). The treatment groups did not differ significantly at baseline with respect to age, gender or education level (Table 1) or HAD-A and D, SF-36 MCS and SOC scores (Table 2) (Figure 2).

**Table 2:** HAD anxiety, HAD depression and SF-36 MCS and Sense of coherence scores (SOC) at baseline, after eight weeks of treatment and 24 weeks from baseline and change between baseline and follow up for Integrative Treatment (IT), Therapeutic acupuncture (TA) and Conventional Treatment CT

	**IT (n = 40)**			**TA (n = 40)**			**CT (n = 40)**						
	**M**	**Mdn**	**SD**	**M**	**Mdn**	**SD**	**M**	**Mdn**	**SD**	**IT/TA/CT *p***^**1**^	**IT/CT *p***^**2**^	**TA/CT *p***^**2**^	**IT/TA *p***^**2**^
HAD Anxiety													
Baseline	9.8	9.5	5.2	11.6	12.0	4.7	11.7	12.0	4.6	.197			
8 wks	5.4	5.0	4.0	6.9	6.5	4.1	10.4	11.0	4.4	**.001**	**.001**	**.001**	.093
Δbas/8wks	4.4	3.5	4.9	4.7	5.0	3.8	1.3	1.0	4.3				
Δ p-value^3^	**.001**			**.001**			.070						
24 wks	5.1	4.5	4.1	7.1	7.1	4.3	8.7	8.7	3.5	**.001**	**.001**	.046*	**.019**
Δbas/24wks	4.7	3	4.4	4.5	3.0	4.2	3.0	3.0	4.3	.080			
Δ p-value^3^	**.001**			**.001**			**.001**						
HAD Depression													
Baseline	7.6	7.0	3.6	7.7	8.0	4.2	7.5	8.0	4.2	.991			
8 wks	3.8	4.0	2.7	4.1	4.0	3.0	7.5	7.5	3.7	**.001**	**.001**	**.001**	.625
Δbas/8wks	3.8	3.0	3.6	3.4	3.0	3.9	0.0	0.0	3.4				
Δ p-value^3^	**.001**			**.001**			.080						
24 wks	3.6	3.6	3.6	4.4	3.0	3.9	6.2	7.0	3.9	**.001**	**.001**	**.011**	.260
Δbas/24wks	4.0	4.0	3.9	3.3	3.0	3.9	1.4	1.0	4.4	.250			
Δ p-value^3^	**.001**			**.001**			.030*						
SF36: MCS													
Baseline	27.6	24.5	12.4	28.5	31.3	13.6	27.1	27.6	12.4	.896			
8 wks	46.1	46.1	9.4	43.4	43.6	11.9	32.7	32.4	14.1	**.001**	**.001**	**.001**	.476
Δbas/8wks	18.5	11.8	18.4	14.9	12.3	11.9	5.5	13.6	5.3	**.001**	**.001**	**.002**	.160
Δ p-value^3^	**.001**			**.001**			.013*						
24 wks	44.6	46.3	11.2	42.6	42.6	12.9	34.6	34.6	12.3	**.001**	**.001**	**.001**	.513
Δbas/24wks	14.0	11.0	15.4	17.0	15.8	13.3	7.5	6.4	15.7	.013*			
Δ p-value^3^	**.001**			**.001**			**.004**						
SOC-13													
Baseline	55.4	56.0	13.7	52.2	54.0	11.6	53.1	51.5	13.8	.533			
8 wks	68.1	68.0	9.6	64.8	65.0	10.4	56.3	56.4	13.3	**.001**	**.001**	**.001**	.954
Δbas/8wks	12.7	11.4	10.0	12.5	10.9	11.0	3.2	11.5	2.5	**.001**	**.001**	**.001**	.951
Δ p-value^3^	**.001**			**.001**			.091			**.001**			
24 wks	65.7	65.9	11.1	64.5	64.5	11.9	58.5	58.5	10.6	**.001**	**.001**	**.004**	.531
Δbas/24wks	10.3	8.7	13.9	12.4	12.0	12.2	5.4	5.5	14.2	.078			
Δ p-value^3^	**.001**			**.001**			**.012**						

HAD: Hospital Anxiety and Depression scale; SF-36: 36-item Short Form health survey; MCS: Mental Component Summary score; SOC: Sense of coherence. ^1^Nonparametric Kruskal-Wallis test, ^2^Mann-Whitney-*U* test, ^3^Wilcoxon signed rank test, p-values less than 0,05 are shown in bold. *Bonferroni correction p < .012

P values for between-group comparisons of baseline scores, 8-week and 24-week scores and baseline to 8-week and 24-week change (Δ), as well as within-group change are shown.

### Within group change between baseline and 24 weeks

As shown in Figure 1, the general pattern for all variables was that the TA and IT groups substantially improved from baseline to 8 weeks and leveled at 24 weeks; whereas the CT group showed slight monotonic improvement from baseline.

**Figure 3:**
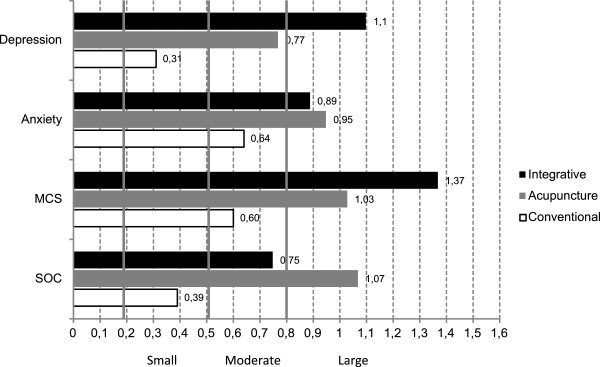
Effect sizes for HAD anxiety, HAD depression, SF-36 MCS and SOC between baseline and 24-week follow up by treatment group.

Compared to baseline values, significant improvement (p > 0.001) was observed on all variables at 24 weeks in TA and IT (Table 2). Similar improvements were noted for CT on all variables (SOC p = 0.012; HAD-A p = 0.001; MCS p = 0.004) except HAD-D; however, no significant improvements were observed in this group between baseline and 8 weeks.Effect sizes were all large in TA and IT for all variables: SOC TA = 1.07, CI = .58-1.51 vs. IT = .75, .36-1.27; HAD-D TA = .77, CI = .37-1.28 vs. IT = 1.10, CI = .64-1.58; HAD-A TA = .95, CI = .52-1.45 vs. IT = .89, CI = .53-1.45; MCS TA = 1.03, CI = .58-1.52 vs. IT = 1.37, CI = .93-1.92), whereas in CT effect sizes were small (SOC = .39, CI = .01-.88; HAD-D = .31, CI = -.11-.78) to moderate (HAD-A = .64, CI .25-1.16; MCS = .60, CI = .15-1.05) (Figure 3).

### Between group differences at 24 weeks

The same pattern of results seen at 8 weeks was also seen at 24 weeks, i.e. both TA and IT had significantly better values (p > 0.05) than CT on SOC, SF-36 MCS and HAD-D and A. After correcting for multiple comparisons (Bonferroni correction: p = 0.05/4 comparisons; p > 0.013), the difference between TA and CT for HAD-A was no longer significant at 24 weeks.

### Between group change between 8 and 24 weeks

No significant differences were seen between 8 and 24 weeks on any variable in TA or IT; however, in CT a significant decrease (p = 0.009) was seen in HAD-A.

### Between group change between baseline and 24 weeks

**Figure 4:**
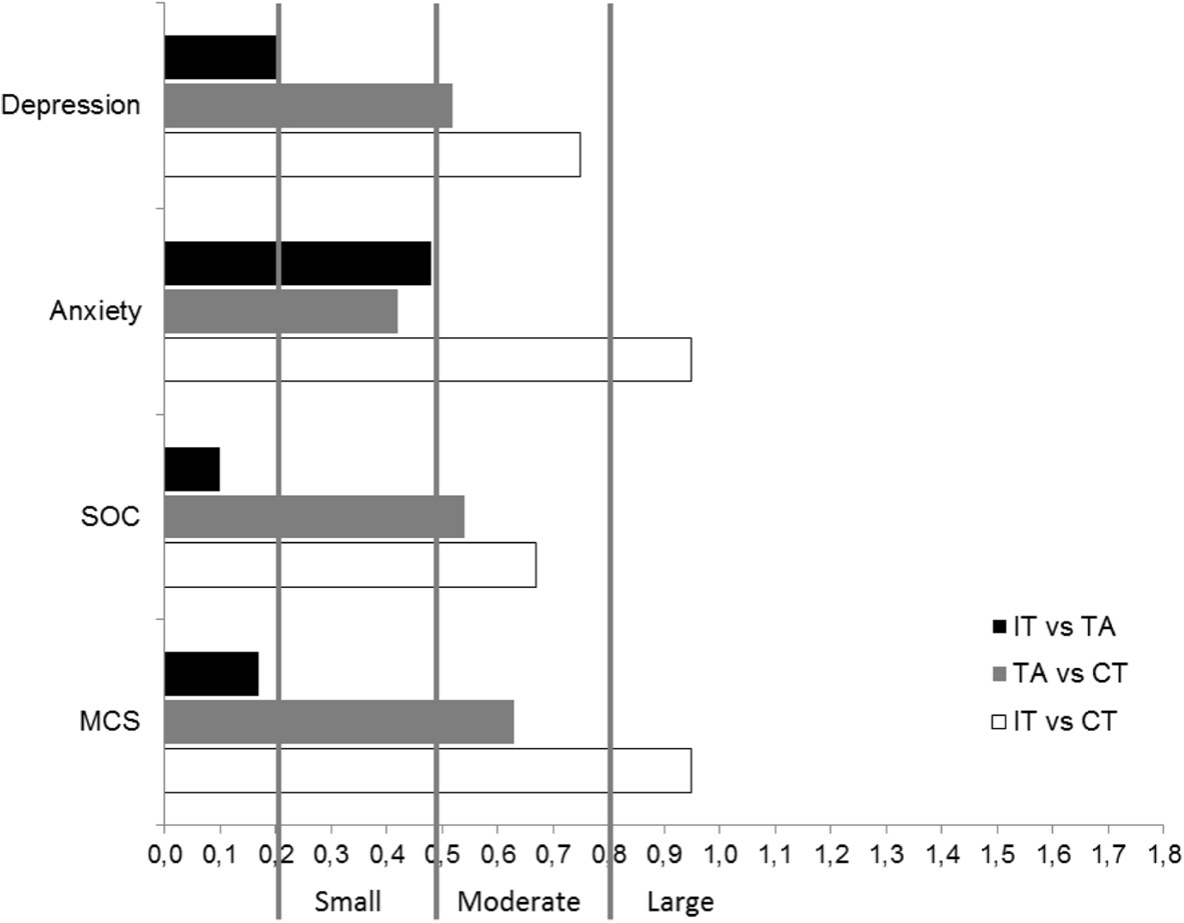
**Effect sizes (ES) comparing integrative treatment (IT), therapeutic acupuncture (TA) and conventional treatment (CT) regarding HAD depression and anxiety, Sense of Coherence (SOC) and Mental Component Summary (MCS) scores at 24 weeks.** ES reflects the magnitude of the effect of the first group relative to the second.

No statistically significant differences were seen between the three groups on any variable concerning the magnitude of change from baseline, after correcting for multiplicity. However, as shown in Figure 4, effect sizes comparing IT and CT were large for HAD-A (ES = .95; CI = .48-1.41) and MCS (ES = .95; CI = .39-1.30) and moderate for SOC (ES = .67; CI = .21-1.11) and HAD-D (ES = .75; CI = .29-1.19). Effect sizes comparing TA and CT were slightly lower, with moderate effect sizes for SOC (ES = .54; CI = .09-.98), HAD-D (ES = .52; CI = .07-.96) and MCS (ES = .63; CI = .18-1.07) and small for HAD-A (ES = .42; CI = -.03-.86). Effect sizes comparing IT and TA were trivial to small (SOC = .10, CI = -.34-.54; MCS = .17, CI = -.21-61; HAD-D = .21, CI = -.23-.65; HAD-A = .48; CI = .03-.92).

### Dropouts

In total, 3 IT, 6 TA and 9 CT patients dropped out during the study period. All 3 IT patients dropped out before eight weeks; 4 of the 6 TA patients dropped out before eight weeks and two before 24 weeks; and 5 of 9 CT patients dropped out before eight weeks and four before 24 weeks (Figure 1). Reasons for dropout were pregnancy, personal reasons and pneumonia in the IT group; personal reasons (n = 3) and no stated reason (n = 3) in the TA group; and no stated reason (n = 9) in the CT group.

## Discussion

This 6-month follow up of an open, pragmatic, randomized controlled trial comparing the effects of an integrative treatment, therapeutic acupuncture and conventional treatment in alleviating psychological distress in primary care patients [25] showed that improvements in anxiety, depression, mental health status and sense of coherence seen immediately after acupuncture and integrative treatments were maintained six months after initiating treatment. Although CT showed gains on all variables from baseline, at six months IT still had significantly better scores on all variables. Effect sizes compared to CT were moderate for sense of coherence (.67) and depression (.75) and large for anxiety (.95) and mental health status (.85). Likewise, TA had significantly better scores than CT on all variables except anxiety, but effect sizes were slightly lower than those between IT and CT (small for anxiety (.42) and moderate for sense of coherence (.54) and depression (.52) and mental health (.63). Furthermore, effect sizes calculated between baseline and six months were large for all variables in IT and TA, whereas CT was associated with small to moderate effect sizes. As was seen at 8-weeks [25], no differences were found between IT and TA on any variable at 6-month follow up.

Our results are in line with earlier findings suggesting the effectiveness of acupuncture in reducing anxiety [19-21] and depression [14,19,23,40]. We have previously shown that both integrative treatment combining therapeutic acupuncture with structured salutogenic dialogue or therapeutic acupuncture with non-directive dialogue reduce depression and anxiety significantly more than usual care in the short term [25]; however, to our knowledge only one randomized controlled study has evaluated longer term effects of acupuncture compared with usual care in alleviating psychological distress [15]. In that study, also conducted in a primary care setting, acupuncture was found to reduce depression significantly more than usual care at 3-month follow up, with a standardized mean difference (SMD) of .39 (CI = .19-.58). In our study, we obtained a larger effect size for acupuncture compared to usual care (ES = .52; CI = .07-.96) and a considerably larger one for integrative treatment versus usual care (ES = .75; CI = .29-1.19). These results may be compared with short term effects reported in a recent Cochrane review [23] corresponding to a SMD of .73 (CI = .29-1.18) favoring manual acupuncture versus waiting list in reducing depression. Differences in the magnitudes of the effects found in these studies may naturally owe to differences in the severity of depression in the samples, length of treatment, timing of follow up, depression assessment instruments, acupuncture methods, etc. However, together these studies suggest that acupuncture may be more effective than usual primary care in alleviating depression in both the short and longer term. Furthermore, by assessing a broader range of outcomes, our study was able to show that acupuncture and acupuncture integrated with non-directive salutogenic dialogue also had significantly greater effects on anxiety, sense of coherence and health-related quality of life at 6-month follow up compared with usual care. Similar positive results for acupuncture versus controls in relation to depression, anxiety and health-related quality of life have recently been reported [40].

We are aware of no other study that has examined the potential added value of an integrative treatment combining therapeutic acupuncture with structured salutogenic dialogue. Based on previous research indicating that salutogenic talk-therapy may be helpful in increasing coping in people with mental health problems [41,42], we hypothesized the potential effects of acupuncture in relieving psychological distress would be strengthened by integrating acupuncture with salutogenic dialogue. Although effect sizes were larger between the integrative treatment and usual care than between acupuncture and usual care at 6-month follow up, no significant differences were seen between the integrative treatment and acupuncture on any variable and effect sizes were trivial (SOC and SF-36 MCS) to small (anxiety and depression). We found similar results in our short term follow up [25]. Hence, we were unable to find evidence pointing to advantages of integrative treatment over acupuncture alone. A possible explanation may be that the same therapist provided both treatments, therefore contaminating treatments.

As illustrated in Figure 2, the effects of both acupuncture and integrative treatments in relation to all outcome variables remained stable during follow up, whereas conventional care showed slight but continued improvement. Although both acupuncture and integrative treatments were associated with significantly better outcomes than conventional care at 6-month follow up, the magnitude of improvement from baseline was not significantly different between the three groups, contrary to previously observed short term effects [25]. Nonetheless, effect sizes between baseline and six months were large for all variables in both acupuncture and integrative treatments, whereas CT was associated with small to moderate effect sizes. Given that the first two groups received no supplementary treatment during follow up it is reasonable to expect no additional improvement during this period. On the other hand, the observed improvement in outcomes for conventional care may owe to delays in treatment initiation, longer intervals between treatment sessions, longer periods for treatment effects to manifest, etc. Similar results have been reported for usual care compared to acupuncture after 9 and 12 months of follow up [15]. Furthermore, conventional treatment comprised a variety of different treatments ranging from watchful waiting to pharmacological, psycho-educational and cognitive behavioral therapies. Sample size limitations prohibited subgroup analyses of specific therapies in this group and hence our results for this group may be seen to reflect only those for “usual” primary care treatment of psychological distress. It may also be speculated that outcomes favoring acupuncture and integrative treatments over conventional care owe to a care effect, i.e. better outcomes resulted from patients receiving more frequent care independent of treatment regimen. Adding a fourth treatment group receiving only the salutogenic dialogue would have enabled us to tease out the effects of care as well as to better differentiate between the effects of acupuncture and the dialogue. Larger and better designed studies are needed to evaluate possible benefits of combining salutogenic dialogue with acupuncture.

Although Antonovsky originally posited that sense of coherence remains stable during adulthood [26], more recently a number of prospective studies have shown that it may vary [43-45]. Our results are in line with the latter studies in indicating that sense of coherence may in fact be changeable and malleable, and may therefore have important implications for the treatment of psychological distress. Helping patients to develop a sense that their life is comprehensible, meaningful and manageable may improve their ability to cope with adversities by enabling them to choose and apply more appropriate coping strategies for a specific stressor [30,35]. As sense of coherence has been shown to be correlated with depression [46] and HRQL [43], assisting patients to gain a sense of coherence may be beneficial for reducing psychological distress and improving wellbeing.

There are a number of limitations to this study. As mentioned, the same acupuncturist performed acupuncture in both treatment groups. Although having the advantage of controlling for therapist effects, this crossed design may blur distinctions between treatments [47]. Secondly, most (84%) patients were women. As women are generally more likely to discuss mental health problems with their physician [48] they may be more likely to be referred for treatment for these problems. Thirdly, detection of statistically significant differences between treatment groups may have been compromised by the fact that the study was likely underpowered. Based on recently published estimates of minimal important differences for the primary outcome (HAD anxiety and depression) [49] a sample size of about 60 patients per treatment arm would have been required (instead of 40). However, effect sizes used here to reflect the magnitudes of mean differences are, unlike significance tests, relatively independent of sample size. Nonetheless, the relatively small sample size prohibited sub analyses to identify subsets or characteristics of patients who would benefit most from the individual treatments. Fourthly, the sample was heterogeneous regarding primary diagnosis; however, it was homogeneous in the sense that patients were referred for complaints of psychological distress. Finally, although follow up took place 6 months after treatment initiation this is still a relatively short follow up period. Studies are needed to assess and compare the long term effects of acupuncture, integrative treatment and usual primary care in alleviating psychological distress.

## Conclusions

Mental health problems are prevalent in the community and primary health care currently has difficulties with their detection, diagnosis and treatment [8]. As patients with psychological distress often prefer complementary and alternative medicine (CAM) modalities for managing psychological distress, it is of importance to evaluate and compare the effects of such treatments relative to usual care both in the short term and long term [50]. The integrative treatment, combining therapeutic acupuncture with structured salutogenic dialogue, and therapeutic acupuncture appear to be well accepted and to have greater intermediate-term effects than usual primary care in reducing anxiety and depression and improving sense of coherence and health-related quality of life. The short term effects of these treatments were large and remained stable over follow up, whereas significant effects of usual care were manifested only at follow up. Hence, the integrative and acupuncture treatments appear to also be more efficient than usual care in alleviating psychological distress. Larger and longer term studies are needed to evaluate the added value of the integrative treatment over therapeutic acupuncture alone and in relation to usual care practices in primary care.

## Competing interests

The authors declare that they have no competing interests.

## Authors’ contributions

TA and CT contributed to planning the study. TA collected data. TA and CT analysed the data. TA, BM and CT authors contributed to the writing of the manuscript. All authors read and approved the final manuscript.

## Pre-publication history

The pre-publication history for this paper can be accessed here:

http://www.biomedcentral.com/1472-6882/14/210/prepub
